# Neuroembryology and functional anatomy of craniofacial clefts

**DOI:** 10.4103/0970-0358.57184

**Published:** 2009-10

**Authors:** Ember L. Ewings, Michael H. Carstens

**Affiliations:** Division of Plastic and Reconstructive Surgery, Department of Surgery at Saint Louis University School of Medicine, Saint Louis, MO

**Keywords:** Cleft, Embryology, Tessier

## Abstract

The master plan of all vertebrate embryos is based on neuroanatomy. The embryo can be anatomically divided into discrete units called neuromeres so that each carries unique genetic traits. Embryonic neural crest cells arising from each neuromere induce development of nerves and concomitant arteries and support the development of specific craniofacial tissues or developmental fields. Fields are assembled upon each other in a programmed spatiotemporal order. Abnormalities in one field can affect the shape and position of developing adjacent fields. Craniofacial clefts represent states of excess or deficiency within and between specific developmental fields. The neuromeric organization of the embryo is the common denominator for understanding normal anatomy and pathology of the head and neck. Tessier's observational cleft classification system can be redefined using neuroanatomic embryology. Reassessment of Tessier's empiric observations demonstrates a more rational rearrangement of cleft zones, particularly near the midline. Neuromeric theory is also a means to understand and define other common craniofacial problems. Cleft palate, encephaloceles, craniosynostosis and cranial base defects may be analyzed in the same way.

## INTRODUCTION

Successful treatment of congenital craniofacial defects relies on a thorough understanding of the embryologic processes leading to their development. Clues to the mechanisms underlying these anomalies lead to an intersection of comparative anatomy, developmental anatomy, neurobiology, genetics, and cleft surgery.[1,2] As progress has been made in each of these fields, over the past 20 years, new concepts in cleft embryology and new treatment strategies based on these fundamentals have been evolving.

Central to the study of the embryologic development of congenital clefts is the concept “the brain predicts the face,” the inverse of DeMeyer's original principle “the face predicts the brain” in understanding holoprosencephaly.[3] That is, the developing embryologic nervous system may be seen as a map from which all subsequent facial tissues are drawn. The elucidation of this neuroanatomy and the pathways in which craniofacial tissues arise or fail to form is the foundation on which we can classify clefts, observe patterns and syndromes, and predict progression with continued facial development.

## Neuromeric Theory of Embryological Development

The human embryo has its own neuroanatomy. The embryonic central nervous system develops in discrete segmental craniocaudal units of neural crest cells called neuromeres.[4–9] The anatomic boundaries of neuromeres are coded for by unique genes; that is, certain combinations of genes are expressed only in a particular zone. These haematic genetic sequences are known as Hox genes.[10–13] The neural crest cells found just outside the neural tube in the domain of a given neuromere will express the same defining set of proteins as those cells within the neural tube. Further, neural crest cells from a given neuromeric level supply specific zones of ectoderm and mesoderm.[14,15] Craniofacial tissues that ultimately develop from these neuromeres can be traced back to their roots by their unique genetic markers.[16–18] As such, the embryologic nervous system can be seen as the master integrative agent of development.

**Figure 1:**
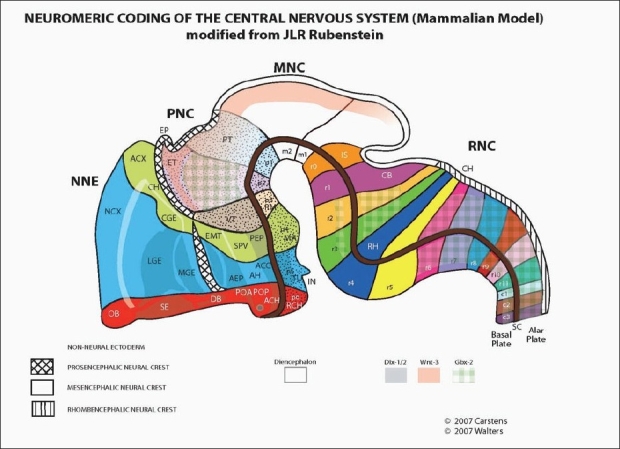
The neuromeric organization of the human embryo. Reproduced from Rubenstein, 1994

The vertebrate central nervous system is divided into three classes of neuromeres[19–22] [Figure 1]. The forebrain is formed from six prosomeres. From caudal to cranial these are numbered p1 to p6. They are subdivided into two tiers, dorsal (alar) and ventral (basal). The telencephalon forms from the alar tiers of p6 and p5. The basal tier of p6 relates to the olfactory system, while basal p5 is associated with the visual apparatus. Puelles and Rubenstein propose that the midbrain is constructed from two mesomeres, m1 and m2. These contain, respectively, the superior and inferior colliculi (an anatomical boundary between the two has not been demonstrated, as it is in the borders between the rhombomeres). The hindbrain is made up of 12 rhombomeres, numbered r0 to r11. An alternative viewpoint held by Samat (personal communication, 2003) considers r0 the principal neuromere of the midbrain and rl the neuromere of the isthmic region (metencephalon, from which develops the pons and cerebellar cortex). Neural crest from r0 and rl (the two mesencephalic neuromeres) is involved in the formation of the orbit. The remainder of the hindbrain (myelencephalon) is made from rhombomeres r2 through r11. These form the medulla. Neural crest originating from neural folds associated with rhombomeres r2-r11 supplies the pharyngeal arch system.

**Figure 2:**
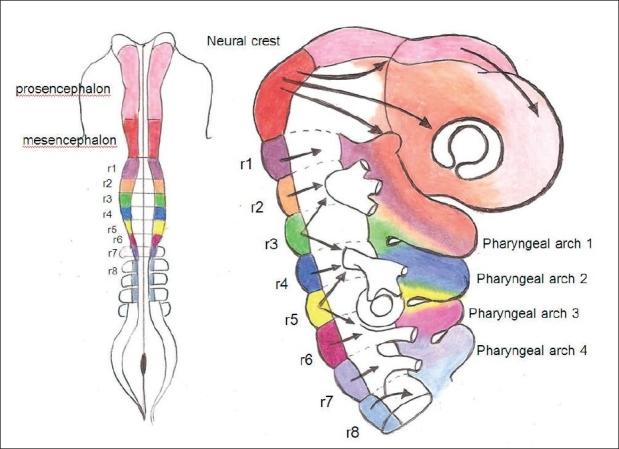
Patterns of neural crest migration into adjacent paraxial mesenchyme and transition into pharyngeal arches. In the forebrain, neural crest travels as a sheet of cells that slides over the more rostral neural folds. In the midbrain, the neural crest cells move in a sequence of streams. In the rhombencephalon, neural crest moves in a segmental fashion laterally into the mesoderm adjacent to the neural tube. Adapted from LeDouarin, 1999

**Figure 3:**
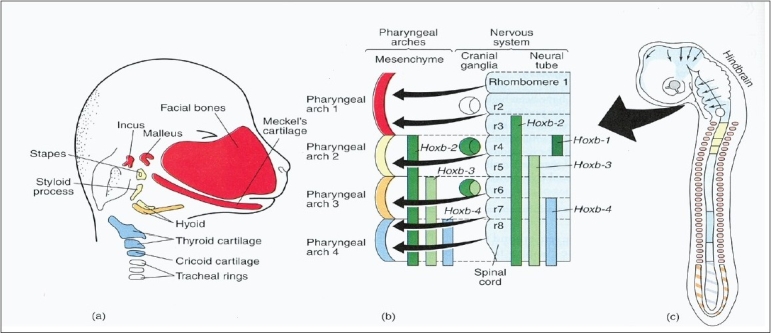
The migration of neural crest cells into the mesenchymal tissues of the pharyngeal arches and subsequent differentiation into the bones of the craniofacial skeleton

When the neural crest cells migrate, they cover the surface of the mesoderm lying just outside the neural tube[23–25] [Figure 2]. In the forebrain, neural crest travels as a sheet of cells that slides over the more rostral neural folds. In the midbrain, the neural crest cells move in a sequence of streams, similar to fighter jets exiting formation one by one. In the rhombencephalon, neural crest moves in a segmental fashion laterally into the mesoderm adjacent to the neural tube. This mesoderm is called paraxial mesoderm (PAM) and is segmented in direct register with the neuromeric system [Figure 3]. Each segment of PAM is called a somitomere (Sm) and is shaped like a ball.[26,27] The first seven somitomeres (corresponding to rl-r7) are incompletely separated. Developmental biologists refer to mesoderm from Sml-Sm7 as cephalic mesoderm. All somitomeres from Sm8 caudally undergo anatomical rearrangement into somites; Sm8-Smll form the four occipital somites and Sml2 becomes the first cervical somite. Therefore, the mesesnchyme of each pharyngeal arch consists of mesoderm from two units of PAM (somitomeres or somites) plus their respective neural crest cells. This mesenchymal tissue, or functional matrix, of the developing human embryo gives rise to the craniofacial skeleton and soft tissues.

As previously mentioned, each segmental unit of the embryo may be identified by unique genetic sequences. During development, these genes express proteins that generally fall into two categories: extracellular signalling proteins and intracellular transcription factors.[27,28] These genes may perform different functions at different times during development, depending on the changing biochemical environment in which they are activated and expressed. In understanding embryonic segmentation and, ultimately, formation of the head, the most important class of transcription factor is the homeodomain proteins.[10,13,29–37] All these have a helix-loop-helix configuration consisting of an identical set of 61 amino acids. The DNA coding for this region, the homeodomain, is a unique sequence of 183 nucleotides known as the homeobox; every single gene producing this type of protein has the same sequence. Because of this molecular anatomy these genes are called homeobox (Hox) genes.

The concept of haematic gene expression was applied to the rhomboencephalon by correlating patterns of gene product expression with cranial nerves, neural crest, and rhombomeres.[38] A Hox ‘barcode’ could be found for rhombomeric levels r3 and levels caudal; similar gene definitions using krox-20 (the human form of this murine gene is EGR2). follistatin, Engrailed and wnt-1 permitted the ‘mapping out’ of rhombomeres r0, rl and r2.[39–41]

The clinical significance of the neuromeric model is that it enables us to map out the anatomical site of origin for all zones of ectoderm and mesoderm supplied by a given zone of the nervous system. The role of neural crest population in those zones, specifically what structures they generate, can also be understood on the basis of their neuromere of origin. The craniofacial skeleton is derived exclusively from neural crest cells,[42,43] with two notable exceptions. The cranial base (basisphenoid and posterior structures) arises from PAM from somitomere 1; the parietal bone is produced by epaxial PAM from somitomeres 2 and 3. The cell population producing the ethmoid, presphenoid, premaxilla, and vomer all originate in antero-posterior order from the neural folds in genetic register with the first rhombomere (rl). The rostral aspect of the second rhombomere (r2) gives rise to the premaxilla and vomer, while the inferior turbinate, palatine bone, alisphenoid, maxilla and zygoma arise from the more caudal neural crest of r2. The squamous temporal, mandible, malleus and incus are r3 neural crest bones. As an example, premaxillary development may be traced (by shared Hox gene sequences) to a precursor cell population in the ‘premaxillary zone’ of mesencephalic neural crest along the neural fold, corresponding to the second rhombomere. A deficiency state in this cell population (inadequate cell number, defective migration, abnormal post-migratory rates of mitosis or cell death) will lead to a small or absent premaxilla. Furthermore, if the premaxillary mesencephalic neural crest has several subsets, aligned in cranio-caudal order along the neural fold (i.e., central incisor, lateral incisor and ascending process), then the spectrum of deficiency states seen in the premaxilla of cleft patients can be understood as progressively greater degrees of disturbance in the premaxillary neural crest precursor population.

The process of neural crest cell migration and differentiation leads to the development of specific craniofacial tissues, as noted above. These zones are referred to as developmental fields. The neural crest cells arising from each neuromere induce the development of nerves and concomitant arteries which in turn support the development of individual fields.[44–46] As the embryo continues to enlarge, develop, and fold, fields migrate forward in tightly regulated spatio-temporal sequence. They cluster around the developing brain and undergo a process of self-assembly. As such, anatomical relationships between fields progress during development. The presence of one field may be required for another field to correctly develop. As such, abnormalities in individual fields may adversely affect the development of otherwise normal adjacent fields. Craniofacial clefts represent a deficiency, excess or absence of an embryonic developmental field, and subsequent impact on surrounding fields.

As noted previously, several mechanisms by which neural crest cell movement is interrupted account for the different types of field abnormalities observed in the developing face. Premigratory losses result from errors within the neuromere of origin. Migratory problems arise when neural crest cell populations die out or get lost on their journey. Postmigratory anomalies stem from faulty interaction between mesenchyme and epithelial “target zone,” or a faulty epithelial “programme.” Thus, inadequate neural crest or mesodermal cell numbers, defective migration, abnormal post-migratory rates of mitosis or cell death, or inability of neural crest cells to induce a supportive neurovascular supply may all contribute to anomalies in the functional matrix available for formation of a given developmental field. Ultimately, the final common pathway to field defects is insufficient induction of vascular support for developing tissues in the given zone. Developmental field anomalies may be divided into five types corresponding to the multiple ways that normal neural crest cell migration, multiplication, and differentiation may be interrupted in the developing embryo. The “invisible” field is observed when an entire developmental field is absent, and the adjacent fields become distorted (as in the Tessier Number 3 cleft, missing inferior turbinate field). Similarly, the “dwarf” field is present but abnormally small, causing deformation and/or restraint of growth of adjacent fields (as in the deficient vomer in an isolated cleft palate). The “dysfunctional family” field represents fields that develop appropriately, but fail to interface correctly with adjacently developing fields, leading to fusion failure at the border zone between them (as in the lateral facial clefts). The “giant” field is larger than normal, pushing adjacent fields aside (as in hypertelorism). Finally, the “leaky” field is seen between normally-developed zones, where a weak suture between them permits brain to escape, and further pushes fields apart (as in encephaloceles).

The appearance of a facial cleft appears differently in the newborn than in the developing embryo, and certainly we have observed the continued progression of deformity in the untreated adolescent and adult with facial clefting. The patterns of deformity are predictable if the spatial development of the embryonic and foetal face is understood. Anatomical changes resulting from developmental field anomalies affect facial morphology in four dimensions of development (“the four ‘D’s” of cleft progression). These dimensions are deficiency, deformation, distortion, and division.[4,47–54] Interestingly enough, the order of these processes follows the order of axis specification in the embryo: anteroposterior, then mediolateral and finally right-left. The pathological sequence of the familiar labiomaxillary cleft illustrates well the developmental progression of these dimensions. First, a deficiency state exists in the functional matrix (mesenchyme) giving rise to the piriform margin, then an abnormal developmental field develops within this insufficient bone volume. This causes a characteristic displacement pattern of the soft tissue envelope on both sides of the cleft. If the deficiency state is significant enough, it affects the ability of adjacent developmental fields to perform soft tissue closure of the nasal floor and lip. The resulting division further aggravates tissue displacement. Over time, the effects of deficiency, displacement and division create a distortion of the overlying soft tissue envelope. This results in an abnormal anatomy of the septum. Ongoing growth of the osteocartilaginous nasal vault, uncoupling of normal relationships between the skeletal elements, and aberrant force vectors exerted by the perioral musculature result in the characteristic ‘opening-up’ of the cleft site so elegantly described by Delaire.[55–60] Thus, each of the “d's” represents a dimension of cleft progression: deficiency is axial; displacement is coronal; division is sagittal; deficiency is temporal.

## Effect of bony defect on overlying soft tissue

In craniofacial development one of the most potent signalling families, the hedgehog proteins, has only recently been isolated.[61–65] In mammals, three forms of this protein (called Sonic hedgehog [SHH], Indian hedgehog and Desert hedgehog) come from three different genes with the same name. In craniofacial development, the function of SHH is to maintain epithelial stability.[66] In order for the facial processes to fuse as they come into proximity to each other, there must be epithelial breakdown at their leading edges.

First, it must be understood that the facial soft tissues of the developing embryo induce the formation of underlying bone. However, genes expressed during bone synthesis reciprocally affect the fusion of overlying soft tissues. Interaction between the SHH present in the soft tissues and bone morphogenic protein type 4 (BMP4) produced by the underlying bone is the key to the relationship between bone and soft tissue clefts. Specifically, the presence of BMP4 during bone formation sends a chemical “signal” to the overlying epithelium, repressing the normal stabilizing function of SHH.[67,68] The epithelium in this region destabilizes and breaks down by the process of cellular apoptosis, allowing for fusion of the facial soft tissues. If a bony cleft is present, a local reduction in BMP4 will lead to persistent epithelialisation of the facial processes an inability to fuse. A soft tissue cleft will be the result.

## Tessier System of Orofacial Clefting

The clinical observations made by Paul Tessier[69–71] regarding patterns of craniofacial cleft formation were derived from empiric observations, but actually match closely patterns of neural crest migration. By examining these pathologic phenomena using the neuroembryologic and genetic knowledge of clefts, a new understanding of the Tessier system shows is based on embryology, rather than topography. Specifically, the developmental fields involved in the areas where the numbered Tessier clefts fall have been mapped out [Table 1]. Abnormalities in the functional matrices giving rise to these fields, or the neurovascular supply supporting them, may lead to the very same clinical malformations observed by Tessier.

**Table 1:** Neuromeric origins and developmental field defects of the Tessier craniofacial clefts

*Tessier Zone*	*Neuromere of Origin*	*Developmental Field*	*Neurovascular Supply*
0	N/A	Fusion failure	N/A
1	r2′	Premaxilla- central incisor	Medial sphenopalatine
2	r2′	Premaxilla-cental, lateral incisors/frontal process	Medial spenopalatine
3	r2	Maxilla, palatine bone, inferior turbinate	Lateral sphenopalatine
4	r2	Medial maxilla	Anterior superior alveolar
5	r2	Middle maxilla	Middle superior alveolar
6	r2	Posterior maxilla	Posterior superior alveolar
7	r2	Jugal	Zygomaticofacial
8	r2	Postorbital	Zygomaticotemporal
9	r2	Alisphenoid	Middle meningeal, anterior deep temporal
10	p5	Postfrontal	Supraorbital
11	p5, r1	Prefrontal, lacrimal	Supratrochlear, dorsal nasal
12	p5, r1	Ethmoid labyrinth	Anterior/posterior ethmoid, lateral nasal branches
13	p5, r1	Ethmoid cribiform	Anterior/posterior ethmoid, medial nasal branches
14	N/A	Fusion failure	N/A

To illustrate the neuromeric theory of embryological development in the Tessier system, each field anomaly will be explored individually. Several common themes underlie the collective series of clefts. First, all of the Tessier cleft zones faithfully follow the neuroanatomy of V1 and V2, along with concomitantly-derived vascular supply. Second, several of the topographically-numbered clefts of the original Tessier classification may at first seem to overlap the same developmental field. When examined in more detail, it can be seen that indeed, each zone corresponds to unique neurovascular anatomy. Futhermore, several zones can be grouped together according to their embryologic beginnings. Zones 4-9 are simplistic, marked by single bones with clearly identified fields (maxilla, zygoma). Zones 10-11 are laminated, in that they are derived from two epithelial layers (dura-sclera, dermis) which interact with an intervening layer of mesenchyme. This causes a split into two laminae, with the resultant formation of sinus cavities (frontal, ethmoid sinuses). Zones 12-13 may be seen as “stacked:” r1 (ethmoid) fields lie internal to p5 (frontal) fields. Thus, it will be demonstrated that zones 1-2 clefts are also manifestations of pathology in zones 12-13. Similarly, zone 0 does not exist as a distinct cleft. Rather, the absence of midline structures results from insult to the internally-situated precursor, the ethmoid. Therefore, midline hypotelorism is an extension of pathology in zone 13, while midline “cleft” hypertelorism results from insults to an external midline approximation mechanism, rather than intrinsic flaws in the tissues.

## Cleft zones 1-9

The clefts in Tessier zones 1 through 9 are all organized around the sensory branches of the maxillary branch of the trigeminal nerve, V2. The pterygopalatine ganglion is the key structure. The “simple” series of infraobital clefts (Tessier Numbers 1-6) are organized around solitary mesenchymal fields (r2′ in the case of cleft 1, vomerine defect and cleft 2, premaxillary defect; r2 in the case of the Number 3 cleft involving the inferior turbinate and palatine bone and maxillary clefts 4-6).

**Figure 4:**
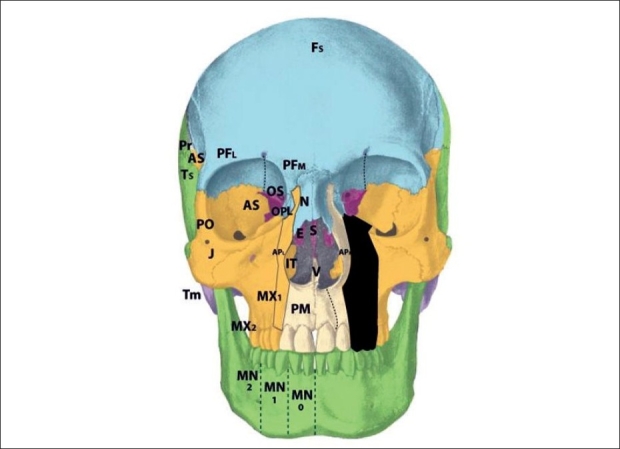
:Tessier zone 1 (premaxillary) cleft, supplied by the medial sphenopalatine artery

The zone 1 cleft represents a defect of the central incisor portion of the premaxilla [Figure 4]. It is mucosal and osseous in composition, and does not include the septum, ethmoid plate, nasal lining, alar/triangular cartilages, or nasal skin (these nasal skin and structures are derived from p5 mesenchyme). Thus, the “nasal notch” is not a feature of the zone 1 cleft, but rather characteristic of the zone 13 cleft. The zone 1 cleft represents field defects of the premaxilla and vomer. In fact, the premaxilla develops before the vomer, and thus the premaxillary fusion to the neighbouring maxillary field fails due to the abnormal premaxillary field. This results in a mechanical deviation of vomer with the premaxillary segment and away from the maxillary segment. A true zone 1 cleft is then observed as an isolated cleft palate.

The mesenchyme giving rise to zone 1 is found at the junction between r1 and r2, in an area designated r2′. This section lies caudal to r1. Thus, a normally developed, pre-existing ethmoid plate (r1) is required for proper formation of the premaxillary segment.

The neurovascular supply to this zone follows the medial sphenopalatine artery. Loss of this branch, or the neural crest cells giving rise to this mesenchyme, may lead to loss of tissues in the zone 1 distribution.

**Figure 5:**
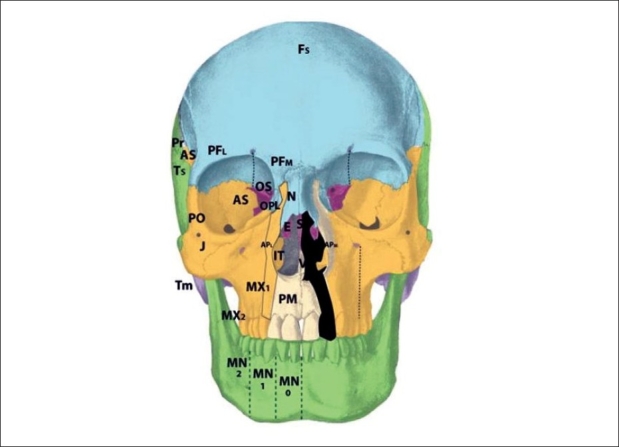
Tessier zone 2 (premaxillary) cleft, supplied by the medial sphenopalatine artery

The premaxilla may be divided into three distinct developmental fields involving the 1) central incisor; 2) lateral incisor and 3) frontal process. During embryological development, the mesenchymal neural crest populates these areas from medial to lateral. The premaxillary fields actually straddles 2 neuromeric (numeric) Tessier zones, with the central incisor belongs to zone 1 and the lateral incisor and frontal processes belonging to zone 2. Thus, the zone 2 cleft represents a field defect of the premaxilla originating from r2′ cranial to the vomer (r2) but caudal to the zone 1 mesenchyme (r2′ caudal to the r1 ethmoid) [Figure 5]. Zone 2 includes the ipsilateral central incisor, lateral incisor, and frontal process. From lateral to medial (more moderate to more severe), loss of the ascending frontal process of the premaxilla results in the reduction of the lateral nasal wall lining directly in front of the inferior turbinate. This results in an overall airway reduction of 30-40%. The piriform aperture is always involved in defects of the frontal process. Loss of the lateral premaxillary segment results in loss of the lateral incisor, an alveolar cleft and frequently (by mechanical displacement of vomer with remaining premaxilla), secondary palatal clefts. Finally, complete loss of the premaxilla including the central portion results in loss of the entire hemi-premaxillary segment. This condition is rare, and is seen in holoprosencephaly. Soft tissues involved are the lip lateral to philtrum; skin and mucosa from r2 (1st arch), and muscles, fat and facial artery derivatives from r4 (2nd arch). The neurovascular supply to zone 2 is derived from the medial sphenopalatine arterial and nerve branches.

**Figure 6:**
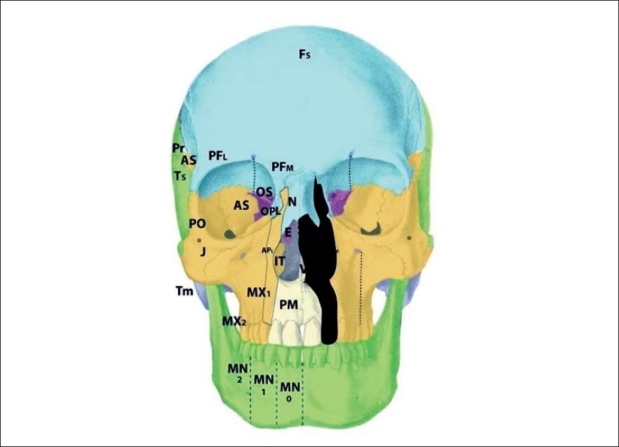
Tessier zone 3 (palatine, maxillary palate, inferior turbinate) cleft, supplied by the lateral sphenopalatine artery

The zone 3 cleft represents a defect spanning three fields: the palatine bone, maxillary palate, and inferior turbinate [Figure 6]. In fact, the lateral nasal wall is comprised of the superimposition of zones 2 and 3. The mesenchymal origin of this zone is from r2. The maxillary palate and palatine bones form at about the same time, and predate the inferior turbinate. Thus, in the zone 3 cleft, the inferior turbinate, which develops later, is deficient while the palatine bone may is present. The palatal cleft observed in zone 3 begins in the palatine bone and spreads posteriorly. This results in a “horseshoe” shaped cleft, with the lateral palatine bone present but falling away medially and posteriorly. The footplate of the lacrimal bone rests on the inferior turbinate, and absence of the turbinate can cause disruption of the lacrimal system. Soft tissue findings include a present but dystrophic medial canthus and lower lid colobomas, medial to the punctum, which remain intact. The deformed canaliculus, however, pulls the normal punctum downward. Disruption of the lacrimal sac leads to tear drainage onto the cheek and propensity toward infection. With concurrent zone 11 involvement, the entire lacrimal system may be destroyed. All skin medial to the nasolacrimal groove is derived from p5 mesenchyme and is supplied by the dorsal nasal arterial branch of the internal carotid artery and innervated by the V1 infratrochlear and lateral nasal nerves.

The nasal ala, derived from p5 mesenchyme, is deformed and retracted upward. The sclera, which arises from r1 neural crest supplied by the nasociliary nerve (V1), and medial lower eyelid dermis from p5 are involved in epibulbar dermoids. Thus, 3-11 clefts involving nasal displacement above the medial canthi demonstrate the relationship to the p6/p5 cribriform plate. The neurovascular supply to the zone 3 cleft is the lateral sphenopalatine artery, which gives off the lateral nasal and descending palatine branches.

**Figure 7:**
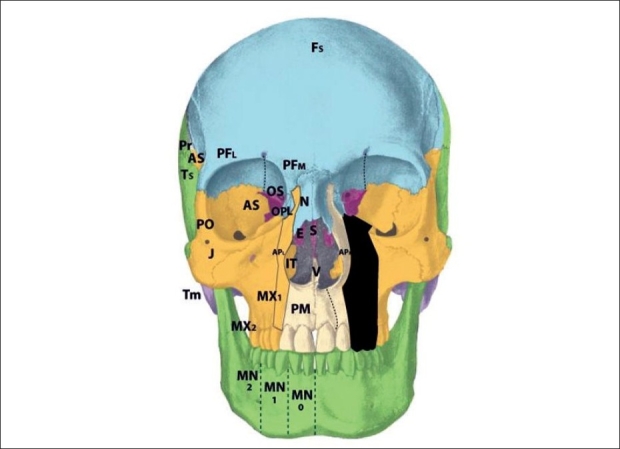
Tessier zone 4 (medial maxillary) cleft, supplied by the anterior superior alveolar artery

The zone 4 defect spans the maxillary wall medial to the inferior orbital foramen and lateral to the lacrimal groove [Figure 7]. The canine is included in this cleft. This tissue is derived from the mesenchyme of r2, and is supplied by the anterior superior alveolar neurovascular bundle, which is an anterior branch of the intramaxillary infraorbital artery and nerve. Zone 4 clefts result in maxillary sinus extrophy, as well as loss of the medial third of the orbital rim and floor. This leads to inferior and medial globe prolapse. This infraorbital musculature is disrupted (meloschesis) secondary to failure of myoblast migration through the cleft in the developing embryo. The punctum, which was present in the zone 3 cleft, is involved in the zone 4 cleft. The nasolacrimal groove, which follows a line from the punctum to the lateral crus of the alar cartilage and is involved in the zone 3 cleft, is medial to the zone 4 cleft and thus is spared. The lacrimal sac is normal but dilated. Deformation of the surrounding normal fields occurs through disconnection of the normal forces between them: the premaxilla becomes protrusive, the lateral nasal walls become retrusive, and the pterygoid plate is displaced forward. Overall, there is an anterioposterior compression of the nasal passages, leading to the clinical appearance of choanal atresia. The zone 4 cleft does not include the piriform fossa or the medial maxillary sinus wall, and the lateral incisor remains intact.

**Figure 8:**
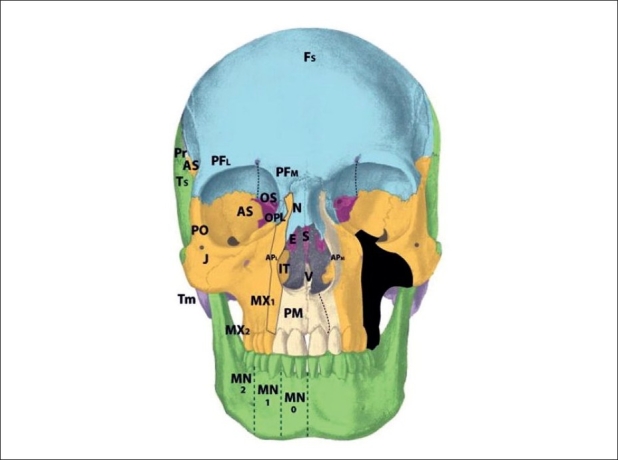
Tessier zone 5 (mid-maxillary) cleft, supplied by the middle superior alveolar artery

The zone 5 cleft represents a defect in the maxilla lateral to the infraorbital nerve [Figure 8]. As a consequence, there is a secondary deformity of zygoma. The premolars are affected, but the maxillary sinus is spared. Soft tissue manifestations include lateral canthal dystopia, involvement of the middle third of the lower eyelid, and cleft lip medial to the commissure but lateral to the typical cleft lip. In fact, in cleft zones 4-6, the axis of the lip defect mirrors that of the lower eyelid. The origin of the zone 5 cleft is in the r2 mesenchyme, caudal to that which gives rise to the zone 4 cleft. The neurovascular axis supplying this zone is the middle superior alveolar branches, which is a posterior branch of the intramaxillary infraorbital nerve.

**Figure 9:**
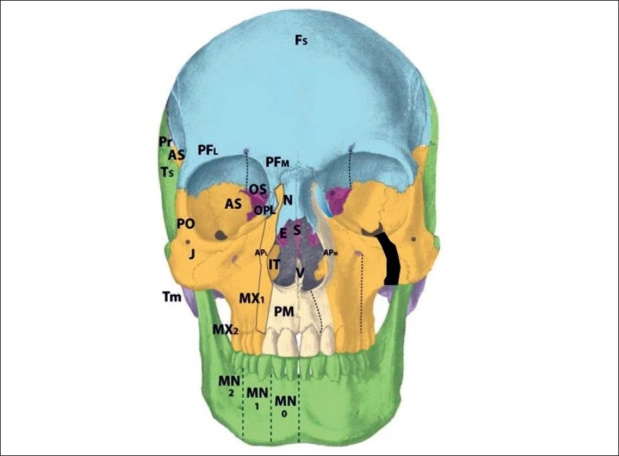
Tessier zone 6 (posterior maxillary) cleft, supplied by the posterior superior alveolar artery

**Figure 10:**
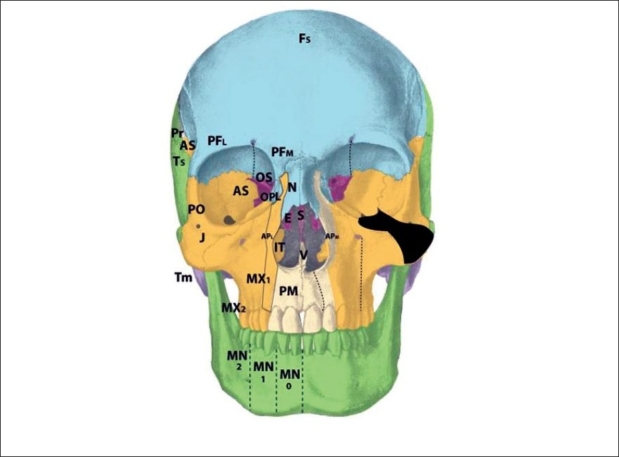
Tessier zone 7 (jugal) cleft, supplied by the zygomaticofacial artery

Clefts in zone 6 represent deficiency of the maxillary wall [Figures 9, 10]. The bony manifestations of the zone 6 cleft include shortening of the posterior maxilla and maxillary buttress; involvement of the inferior orbital fissure; hypoplastic molars with an alveolar “crease,” and a high vaulted palate with posterior choanal atresia resulting from palatine deficiency. The zygomatic arch is not affected. The soft tissue features of the zone 6 cleft are colobomas of the lateral one-third of the lower eyelid with downward displacement of the lateral palpebral fissure and lateral canthus; vertical furrowing of the lateral eyelid to the commissure in cases if incomplete clefting, and macrostomia.

The mesenchymal origin of the zone 6 cleft is r2. The neurovascular supply to this zone is from the posterior superior alveolar branches, with collateral from the ascending pharyngeal artery and nerve.

## Sidewall series of laterofacial clefts

The “sidewall” series represents a combination of defects resulting from solitary field deficiencies (i.e., jugal bone in the number 7 cleft, postorbital bone in number 8 cleft, and alispenoid in the number nine cleft; all derivatives of r2) as well as clefts involving multiple r2-derived fields. These may involve the mirror-image mandibular (r3) fields as well, and are frequently syndromic and symmetric, as in Treacher-Collins and Goldenhar syndromes. In the syndromic forms, additional pharyngeal arch derivatives may also be involved: facial muscle weakness and parotid absence related to the second pharyngeal arch; microtia and levator veli palatini dysfunction related to the third pharyngeal arch. The neurovascular supply to zones 7-9 is from branches of V2 and the internal maxillary artery.

**Figure 11:**
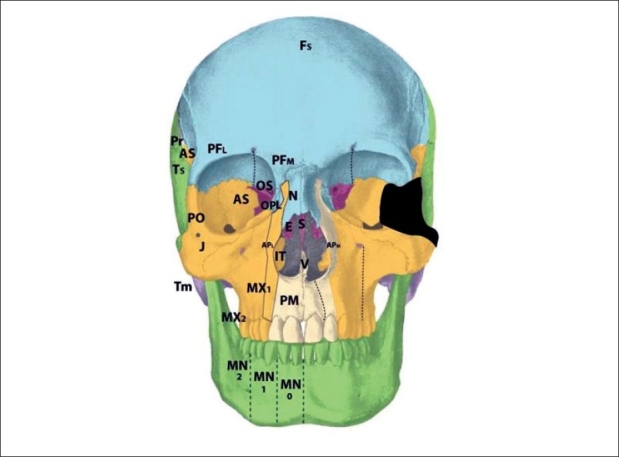
Tessier zone 8 (postorbital) cleft, supplied by the zygomaticofacial artery

The isolated zone 7 cleft demonstrates a deficiency in the jugal bone, or caudal zygoma [Figure 11]. Consequences of the zone 7 cleft include malar flattening and soft tissue clefting from the inferior orbital fissure toward the commissure. The maxilla may show retrusion as a restrained neighbouring field, with Angle Class III occlusion. As compared to the zone 6 cleft, in which the maxillary buttress is absent but the zygoma is present, a zone 7 cleft shows presence of the zygomaticomaxillary buttress but possible involvement of the zygomatic arch. The lateral canthus is displaced downward. Again, the zone 7 cleft is seen as a part of several syndromic conditions, but may exist in an isolated form. This cleft originates from defects in r2 mesenchyme and is supplied by the zygomaticofacial neurovascular bundle.

**Figure 12:**
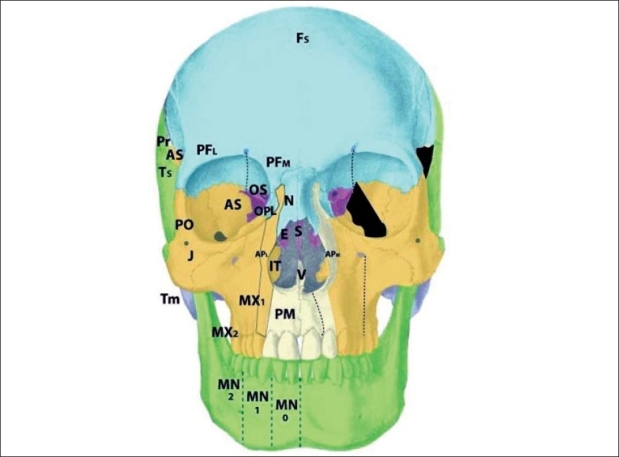
Tessier zone 9 (alisphenoid) cleft, supplied by the zygomaticotemporal and anterior deep temporal arteries

The zone 8 cleft represents a defect in the postorbital bone, marked by the frontozygomatic and sphenozygomatic sutures [Figure 12]. The bony defect observed in the zone 8 cleft is deformity of the greater wing of the sphenoid and lateral orbital wall with downward deformation of the lateral orbital rim. The jugal bone is unaffected. The soft tissue findings include an absent lateral canthus with coloboma and epibulbar dermoid formation and loss of globe support. The zone 8 cleft may again be observed as a component of several craniofacial syndromic conditions. Soft tissue deficiencies predominate in Goldenhar's syndrome whereas ossous defects predominate in Treacher-Collins syndrome. The mesenchyme giving rise to this area is in r2. The neurovascular supply is from the zygomaticofacial branch.

**Figure 13:**
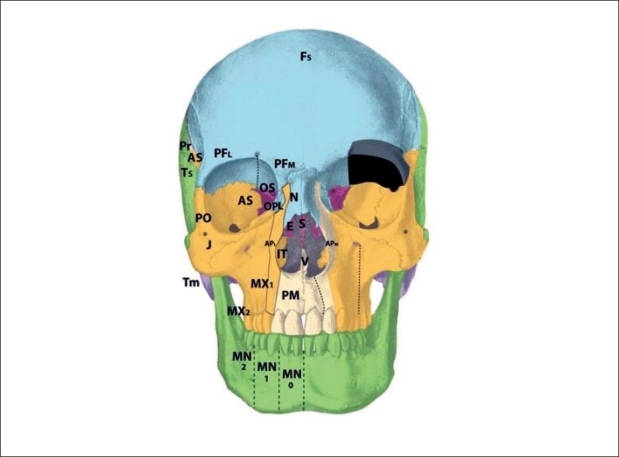
Tessier zone 10 (postfrontal) cleft, supplied by the supraorbital artery

Deficiency of the alisphenoid, or greater sphenoid wing, gives rise to the zone 9 cleft [Figure 13]. The bony defect demonstrates distortion of the neighboring parietal, squamous temporal, and frontal bones. The lateral frontal bone defect leads to entrapment of inferolateral frontal mesenchyme (there is no intrinsic deficiency of p5 frontal mesenchyme). Synostosis of lower coronal suture is observed, as is a hypoplastic lateral pterygoid plate. Soft tissue manifestations include lateral displacement of the globe and colobomata of the lateral third of the upper lid. Additionally, the hairline is distorted with upward displacement of the brow and forward displacement of the temporal hairline and sideburn. This occurs due to mesenchymal deficiency of the r2-derived dermis in this region; reduction in this skin pulls the sideburn forward. Additionally, there may be palsy of the frontalis and upper orbicularis muscles in the region. The zone 9 cleft originates in the r2 mesenchyme, and is supplied by the zygomaticotemporal nerve and anterior deep temporal artery. The zone 9 alisphenoid region is unique in that it exists in a watershed area, at the boundary between the sphenoid and frontal bones (sphenofrontal suture), between the internal carotid arterial systems and external carotid system; between V1-innervated structures and V2-innervated structures; and between p5 (frontal)-derived mesenchyme and r2 mesenchyme. The thick fascial band between the frontalis and temporalis muscles that must be incised during a facelift is a marker of this fusion zone.

## Skull series of craniofacial clefts

The “skull” series of cranial-orbital clefts (Tessier Numbers 10-13) are unique in that there is pathological involvement of multiple interacting fields. The r1 spheno-ethmoid and p5 frontal, nasal and lacrimal fields are all potentially affected by mesenchymal irregularities in any given field. The nerve supply to these regions is from branches of V1. As mentioned previously, the seemingly overlapping neuromeric zones can lead to different anatomical outcomes due to the lamination process, in the case of cleft zones 10-11, and “stacking” that occur in zones 12-14 during the folding and development of the embryo. Any reduction in zones 10-13 will reduce the overall transverse dimension of the anterior cranial fossa, and can lead to hypotelorism. Conversely, excesses in these regions or clefts permitting encephalocele herniation may lead to hypertelorism.

## Cleft zones 10-11

**Figure 14:**
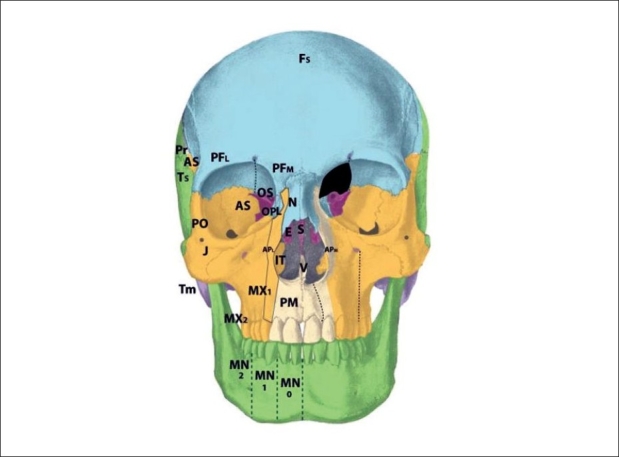
Tessier zone 11 (prefrontal, lacrimal) cleft, supplied by the supratrochlear and dorsal nasal arteries

Zone 10 clefts demonstrate a field defect of the postfrontal region [Figure 14]. This is the area of the orbital roof lateral to the supraorbital nerve and medial to the alisphenoid. The bony defect permits a frontal encephalocele to displace the globe infero-laterally, with distortion of the anterior cranial fossa. Soft tissue findings include coloboma of the middle third of the upper lid; distortion of the middle eyebrow, and downward displacement of the frontal hairline towards the brow. Of interest, findings observed in trigonocephaly also point to pathology in zone 10, rather than simply a single-suture synostosis. Cases of unilateral trigonocephaly demonstrate deficiency in the zone 10 region, and involvement of the p5 frontal mesenchyme more readily explains cases of profound developmental delay than does a single-suture fusion. The mesenchyme giving rise to zone 10 tissues is found in the p5 area of the prosencephalon. The neurovascular axis is the supraorbital artery and nerve.

## Upward Displacement of Ala V1-V2 Boundary from Punctum to Ala = Lacrimal Groove

**Figure 15:**
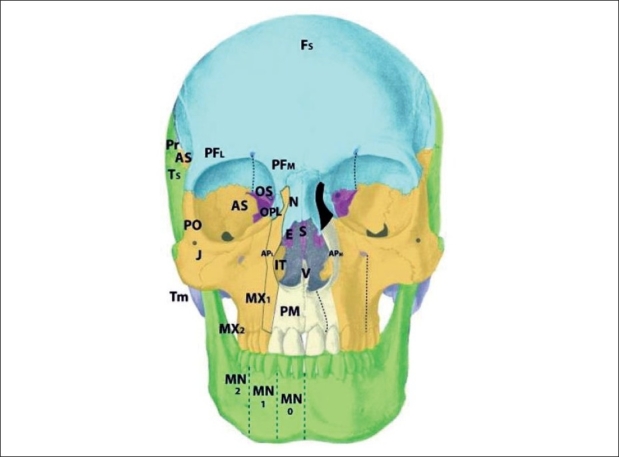
Tessier zone 12(ethmoid labyrinth) cleft, supplied by the anterior and posterior ethmoid lateral nasal artery

The zone 11 cleft represents defects in the prefrontal bone medial to the supraorbital nerve and lateral to the ethmoid, and the lacrimal bone [Figure 15]. The bony loss is of the medial wall of the orbit, as well as the lateral wall of the ethmoid labyrinth. The consequences of the zone 11 cleft include encephalocele through the defective frontoethmoid suture at the lateral border of the cribiform plate. Orbital dystopia results. Coloboma of the medial third of the upper lid may be observed, along with brow and hairline distortion in the same region. A more subtle form of the zone 11 cleft is the isolated lacrimal bone deficiency. Lacrimal stenosis may occur when distorted orbicularis insertions affect the lacrimal pump mechanism. The mesenchymal origin of the zone 11 cleft is in p5 (prefrontal) as well as r1 (lacrimal), and the neurovascular axis is the supratrochlear and dorsal nasal arteries and nerves. In #11: posterior ala is pulled upward, causing inward rotation.

## Cleft zones 12-14

**Figure 16:**
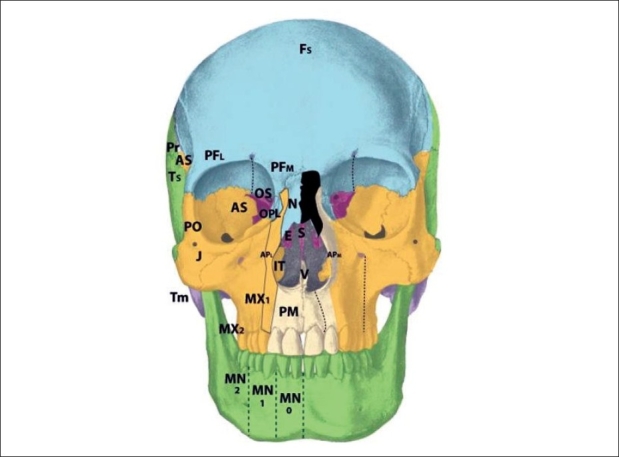
Tessier zone 13 (ethmoid cribriform) cleft, supplied by the anterior and posterior ethmoid (medial nasal) branches and the supratrochlear artery

The zone 12 cleft demonstrates defective fields in the regions of the ethmoid labyrinth and lateral glabella [Figure 16]. The labyrinthine ethmoid is actually enlarged, leading to lateral displacement of the orbits and hypertelorism. There is no eyelid involvement. The neighbouring fields become involved, with lacrimal bone displacement, upward distortion of the maxillary frontal process, and a shortened piriform. The anterior ala derived from r2 mesenchyme forms normally but becomes distorted. This is beacause the anterior alae pulled upward and externally are rotated by forces acting on these soft tissues by the zone 12 pathology. This occurs because the middle third of the alar rim represents the border zone between the p5 mesenchyme and r2 mesenchyme. The mesenchyme that gives rise to the zone 12 field is found in p5 and r1 (embryologically “stacked” during development). The anterior and posterior ethmoid lateral nasal arterial branches and nerves supply the area through the nasociliary axis. The infratrochlear neurovascular bundle supplies the upper lateral nasal skin, medial to the lacrimal duct. The supratrochlear branches supply the forehead skin derived from p5 mesenchyme.

**Figure 17:**
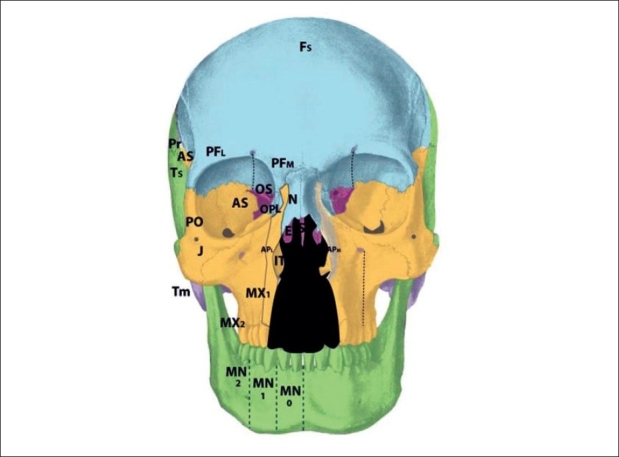
Tessier zone 14 cleft, representing midline fusion failure and/or bilateral zone 12/13 pathology

The zone 13 cleft is marked by field defects of the medial ethmoid cribriform, crista galli, and medial glabella [Figure 17]. There is an excess of tissue in the widened cribiform (r1), an enlarged frontal sinus as well as deficiencies in the nasal bone and ethmoid regions; resulting in hypertelorism. An encephalocele may develop if there is a defective suture between the nasal bone and frontal process of the maxilla, or through a defective suture between the cribiform and orbital roof. The cleft runs through the junction of the p5 nasal skin and r2 alar skin, and results in an alar cartilage cleft at the intermediate crus, without rotation of the alar base. This is in contrast to the previously discussed alar deformities in the zone 11 and 12 clefts, which were consequences of adjacent field pathologies rather than direct involvement. The mesenchyme giving rise to the zone 13 cleft originates from p5 and r1. The neurovascular axes are the anterior and posterior ethmoid (medial nasal) branches and the supratrochlear artery and nerve.

**Figure 18:**
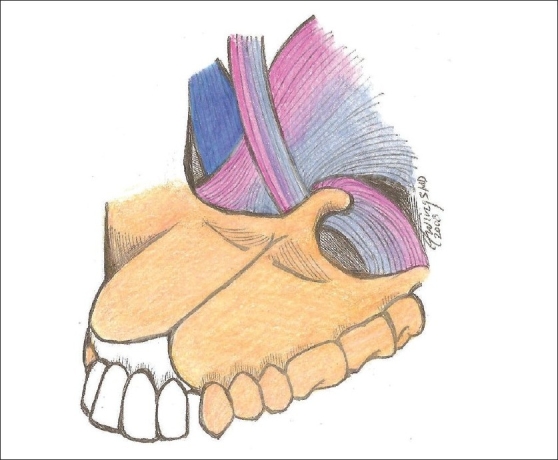
Assembly of the palate from the vomer, premaxillary, and maxillary fields. If the critical contact distance between the premaxilla and maxillaryshelves is exceeded, a primary palatal cleft results. If the critical contact distance between the vomer and maxillary shelves is exceeded, a secondary palatal cleft results. If both distances are exceeded, both primary and secondary palatal clefts result

The zone 14 cleft is unique in that it may represent a number of different clinical manifestations, encompassing the “invisible,” “dwarf,” “leaky,” or “giant” field pathologies, but is not actually a true anatomical “zone.” Rather, the midline deformities are a reflection of either bilateral zone 12-13 pathologies, and/or midline fusion failure. There is deficiency of the ethmoid bone (originating from r1 mesenchyme) as well as excess in the frontonasal region (derived from p5 mesenchyme). The clinical manifestation of ethmoid deficiency is holoprosencephaly, while the excess tissue in the frontonasal zone may be seen as in frontonasal dysplasia. Transverse deficiency of the ethmoid results in an attenuated cribiform plate and labyrinth, with hypotelorism. Vertical deficiency of the perpendicular plate of the ethmoid leads to faulty subsequent premaxillary and vomerine synthesis (from r2′ mesenchyme). An absent perpendicular plate gives rise to bilateral cleft lip and palate, with absence of the premaxillary field [Figure 18]. Alternatively, there may be midline separation of the maxillary shelves, with upward tilting of the palatal planes if the ethmoid/vomer complex is present but shortened. Varying degrees of involvement of the frontal lobe of the cerebrum, which is also derived from p5 mesenchyme, may span the spectrum from isolated absence of the corpus callosum to agenesis of the frontal lobe. In contrast, frontonasal dysplasia (excess) is usually accompanied by normal brain development. Soft tissue manifestations of zone 14 pathology includes encephalocele, nasal dermoids; midline nasal deformities (bifid or hypoplastic), thickened or atrophic nasal skin (due to); midline vermillion notching; and columellar/septal dermoid formation.

## Palatal clefts by mechanism – field theory

Another method of examining the clinical manifestations of the neuromeric theory of cleft development is to review in detail the multiple forms of palatal clefts, with differing embryological bases and potentially differing outcomes. As mentioned previously, several of the named Tessier zones lead to clefting of the palate, though each demonstrated involvement of varied and sometimes multiple fields.

The following is an explanation of how the physical appearance of a single clinical condition (cleft palate) may actually be differentiated into multiple separate etiologies by the neuromeric origins of the involved fields [Table 2]. This allows the clinician to not only understand the embryologic basis of disease, but also gives clues to related anatomy and consequently, predictors of treatment outcomes based on this anatomy.

**Table 2:** Stratification of palatal clefts by mechanism

*Palatal field*	*Neuromeric Origin*	*Cleft Type*
Ethmoid	r1	Unilateral, bilateral, high arched palate, or premaxillary absence
Vomer	r2′	Isolated cleft palate
Premaxilla	r2′	Cleft lip-associated cleft palate; primary +/− secondary palatal cleft
Maxilla	r2	Primary, secondary palatal cleft or both
Palatine bone	r2	Horseshoe-shaped, posterior third
Mandible	r3	Mechanical obstruction from tongue malposition
Tensor veli palatini	r4	Submucous; good pharyngeal wall function
Levator, superior constrictor	r6/7	Submucous; poor pharyngeal wall function
Tongue	r8-11	Mechanical obstruction of palatal closure

**Figure 19:**
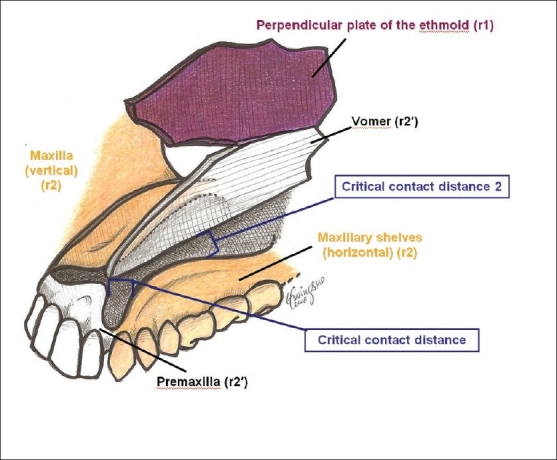
Somitomere 7 pathology (superior constrictor muscle, levator veli palatini) results in muscular dysfunction in a submucous cleft

The palate is built from six discrete developmental fields.[72] Central to the understanding of how the individual fields interrelate is the concept of the critical contact distance in field fusion. Adjacent developmental fields must maintain a precise spatial relationship in order for palatogenesis to succeed. The critical contact distance is the maximum allowable distance between fields, beyond which fusion failure occurs. In the primary palate, anterior to the incisive foramen, fusion must occur between the premaxilla and maxillary shelves. When the premaxilla is deficient, the critical contact distance is exceeded and primary cleft palate results. The vomer may be dragged away from the midline with the untethered premaxilla as premaxillary-maxillary divergence occurs. In the secondary palate, posterior to the incisive foramen, fusion must occur between the lateral maxillary shelves at the midline intersection with the inferior vomer. If the vomer is short, or the shelves are deficient or blocked, secondary palatal cleft results. [Figure 19]

The first palatal field is the ethmoid field, derived from r1 mesenchyme. During normal palatal development, the ethmoid forms first, and the premaxillary and vomerine fields subsequently build on the ethmoid. Therefore, problems with the ethmoid field (specifically, the perpendicular plate) will affect normal development of the vomer and premaxilla. The perpendicular plates of the ethmoid are normally paired and fuse during embryogenesis. Therefore, deficiency will vary depending on whether the pathology is unilateral or bilateral. If one perpendicular plate is gone, a unilateral cleft palate will result, with an unusually small premaxilla and vomer on the non-cleft side. In the case of the bilateral absence of the perpendicular plates, the clinical appearance is of a bilateral cleft lip and palate, with complete absence of the premaxilla. This is a variant of the Tessier zone 14 pathology discussed previously, which manifests as holoprosencephaly. This cleft appearance may be initially deceptive, in that it appears to result from a maxillary defect. In fact, the complete ethmoid deficiency prevents normal development. Additionally, faulty interaction between the r1-derived ethmoid bone and p5-derived frontal mesenchyme may lead to central nervous system dysplasia and developmental delay. There may be associated nasal hypoplasia or agenesis due to the associated p5 mesenchymal abnormality.

Another variation of the r1 ethmoid-derived palatal deformity is the high arched palate. In this instance, the perpendicular plate of the ethmoid is present bilaterally during embryogenesis, but attenuated. The vomer and premaxilla are present, but the horizontal palatal shelves are tilted upwards to meet the midline structures. Thus, the palatal vault depth is increased.

The second palatal field is the vomer field. An isolated vomerine field abnormality will manifest clinically as an isolated cleft palate. This defect arises from a deficiency in the r2′ mesenchyme giving rise to the vomer field. A vertically short vomer is unable to reach the palatal plane, making a prohibitively wide critical contact distance necessary for fusion with the maxillary palatal shelves. The palatal shelves will not fuse at the midline. Where the vomer stops vertically, the cleft palate begins.

The cleft lip-associated cleft palate represents a deficiency of the premaxillary field, derived from r2′ mesenchyme. This corresponds to the Tessier zone 2 cleft palate. With a deficient premaxillary field in the area of the lateral incisor, the critical contact distance between the premaxilla and maxillary shelves is too great to allow for fusion. This is the primary palatal cleft. As the premaxillary segment drifts away from the maxillary segment as embryologic development continues, it carries the vomer away from the maxillary shelves as well. If the vomer diverges too far from the maxillary shelves, fusion will become impossible and a secondary palatal cleft results. Alternatively, the vomerine field may be attenuated as a consequence of the deficient premaxillary field which precedes its development. Thus, the cleft lip can be associated with either a primary palatal cleft; both primary and secondary palatal clefts; and a secondary palatal cleft.

Similarly, deficiency in the r2-derived maxillary shelves may cause horizontally shortened palatal shelves which are unable to meet the normally developing vomer at the midline. The span to reach the vomer exceeds the critical contact distance necessary for fusion, and clefting of the secondary palate results. If the deficiency involves only palatal shelves, a wide cleft of the posterior third of the secondary palate results. If both the maxillary and palatal shelves are involved, a horseshoe-shaped cleft will be observed. The size of the palatine shelf never exceeds the posterior margin of the maxillary shelf. The Tessier zone 3 defect represents deficiency of the maxillary shelf, palatal shelves and additionally, the inferior turbinate. In the Tessier zone 4 cleft, the absent frontal process of the maxilla leads to deficiency of the maxillary alveolus and a primary palatal cleft involving the maxillary canine.

The submucous cleft demonstrates pathology intrinsic to the r6 and r7 mesenchyme residing in the third pharyngeal arch. The superior constrictor muscle (along with the palatoglossus, palatopharyngeus, and uvulus) originates from r6 and r7 in somitomere 7 in the third arch. This muscle works in conjunction with the levator veli palatini (also derived from somitomere 7). Soft palate repair addressing the reorientation of the levator may show disappointing speech outcomes. In this instance, treatment of residual velopharyngeal insufficiency must address the abnormal function of the superior constrictor as well. Pharyngeal surgery, especially sphincter pharyngoplasty, may be required to achieve velopharyngeal competence.

Derived from mesenchyme in somitomere 4, in pharyngeal arch 1, a deficiency in the tensor veli palatini may unleash a developmental sequence leading to submucous cleft palate and complete clefting of the soft palate. Abnormal insertion of the early-developing tensor onto the palate leads to abnormal subsequent migration and insertion of the ensuing third arch palatal musculature. In this case, however, the third arch pharyngeal musculature has normal mesenchymal beginnings and may lead to better functional outcomes than those observed in the circumstance of somitomere 7 pathology.

Somitomeres 8-11 are indirectly involved in palatal closure. Mesenchymal abnormalities arising form these occipital somites are seen as the soft tissue stigmata of Down's syndrome (DS). The misdistribution of mesenchyme in these somites results in an abnormally large myotome (large tongue) and disproportionally small sclerotome (small occiput, low hairline, low-set ears). The enlarged tongue causes a physical obstruction to palatal development, resulting in a high arched palate. Alternatively, the enlarged tongue interferes with the closure of an otherwise normal palate by a mechanical obstruction to midline fusion.

Similarly, mechanical obstruction caused by an abnormally positioned tongue and mandible (derived from r3 mesenchyme) gives rise in to cleft palate in Pierre-Robin sequence. Again, the palatal components are developmentally normal, but physical blockage from an abnormal intervening field results in cleft formation.

To summarize, deficiency states in at least six developmental fields (ethmoid, vomer, premaxilla, maxilla, palatal and pharyngeal musculature, tongue) can lead to cleft palate. Cleft palate pathophysiology may be more accurately categorized by the field(s) involved in their formation. Neuromeric terminology provides an embryologic anatomic basis for differentiating the underlying embryologic mechanism of cleft palate formation, and may lead to a more complete understanding and treatment of associated developmental and functional defects.

## Implications for cleft surgery and management

All surgeons involved with cleft care know fully well the frustration of seeing well executed repairs in infancy degenerate into a predictable sequence of secondary deformities requiring further correction. Even in the best of hands, reoperation rates may reach as high as 85%.[73] What exists here is not failure of technique but an inadequate biological model of the problem in the first place. If the pathological anatomy of the cleft site hinges on a deficiency state in a specific developmental field, and if the surgical correction of the cleft does not include reconstitution of that defective field such that it will grow normally over time and will cease to perturb the growth of its neighbouring fields, then all forms of cleft surgery are condemned over time to varying degrees of relapse. However, in paediatric craniofacial surgery, all patterns of relapse unequivocally indicate the original pathology. Relapse is nothing more than the manifestation over time of the natural anatomical consequences of a deficient developmental field. New approaches to repair of craniofacial clefts that respect and redefine developmental field boundaries may, in essence, “reset” craniofacial growth into a more normal pattern.[45–52]

Neuromeric theory is a means by which to understand and define common congenital craniofacial problems. Encephaloceles, craniosynostosis, microsomia, craniofacial syndromes, and cranial base defects may be analyzed in the same way. The neuromeric organization of the embryo is the common denominator for understanding normal developmental anatomy and pathology of the head and neck.
